# HMGB1 is upregulated in the airways in asthma and potentiates airway smooth muscle contraction via TLR4

**DOI:** 10.1016/j.jaci.2016.11.049

**Published:** 2017-08

**Authors:** Leonarda Di Candia, Edith Gomez, Emilie Venereau, Latifa Chachi, Davinder Kaur, Marco E. Bianchi, R.A. John Challiss, Christopher E. Brightling, Ruth M. Saunders

**Affiliations:** a Institute for Lung Health, Department of Infection, Immunity & Inflammation, Glenfield Hospital, University of Leicester, Leicester, United Kingdom; b San Raffaele University and Scientific Institute and HMGBiotech s.r.l., Milan, Italy; c Department of Molecular and Cell Biology, University of Leicester, Leicester, United Kingdom

To the Editor:

Asthma is characterized by variable airflow obstruction, airway hyperresponsiveness, and inflammation. Airway smooth muscle (ASM) contributes to asthma pathophysiology via hypercontractility, increased mass, and inflammatory mediator release.^1^ Clinical studies and animal models demonstrate a role for high-mobility group box 1 (HMGB1) and its receptors in airway inflammation and asthma.^2^, ^3^ HMGB1's activity and receptor interactions is determined by its redox state, with oxidation rendering HMGB1 inactive.^4^ We have investigated the redox state of airway HMGB1 and the role of HMGB1 in ASM function.

HMGB1 expression and/or redox state was investigated in sputum by ELISA, nonreducing electrophoresis, and Western blotting; in bronchial tissue by immunohistochemistry; and in ASM cells (ASMCs) by quantitative PCR, immunofluorescence, and flow cytometry. The effect of HMGB1 on ASMC reactive oxygen species (ROS) production, migration, proliferation, apoptosis, and contraction was evaluated. Leicestershire Research Ethics Committee approved the study, with informed consent obtained from all subjects. Statistical analysis was performed using GraphPad Prism 6.0. For detailed Methods, see this article's Online Repository at www.jacionline.org.

Sputum HMGB1 concentration was increased in those with severe asthma but not in those with mild to moderate asthma versus healthy controls (controls) (Fig 1, *A*; see Table E1 in this article's Online Repository at www.jacionline.org), correlating significantly with total cell counts and nonviable cell counts/g sputum (see Fig E1, *A* and *B*, in this article's Online Repository at www.jacionline.org), but not sputum differential cell counts (ie, % eosinophils, neutrophils, macrophages, lymphocytes, or epithelial cells) nor lung function (data not shown). HMGB1 concentration in sputum from those with severe asthma was unaffected by oral corticosteroid (OCS) treatment, and did not correlate with OCS dose (data not shown). Both disulphide and reduced HMGB1 were significantly increased in sputum from those with severe asthma versus controls (Fig 1, *B*). In sputum with detectable HMGB1, the proportion of reduced versus disulphide HMGB1 was increased in those with severe asthma versus controls (Fig 1, *C*). Sputum endogenous secretory receptor for advanced glycosylation end products (endogenous secretory RAGE), measured in a subset of sputum samples, was not different between groups (Fig E1, *C*; see Table E2 in this article's Online Repository at www.jacionline.org). HMGB1 expression was significantly increased in ASM in bronchial biopsies from those with severe asthma versus controls (Fig 1, *D*; see Table E3 in this article's Online Repository at www.jacionline.org), with no effect of OCS observed. No differences in ASM RAGE or epithelial HMGB1/RAGE expression in bronchial biopsies were observed (Fig E1, *D-F*; Table E3). Representative photomicrographs of HMGB1/RAGE staining are shown in Fig E1, *G-L*, in this article's Online Repository.

**Fig 1:**
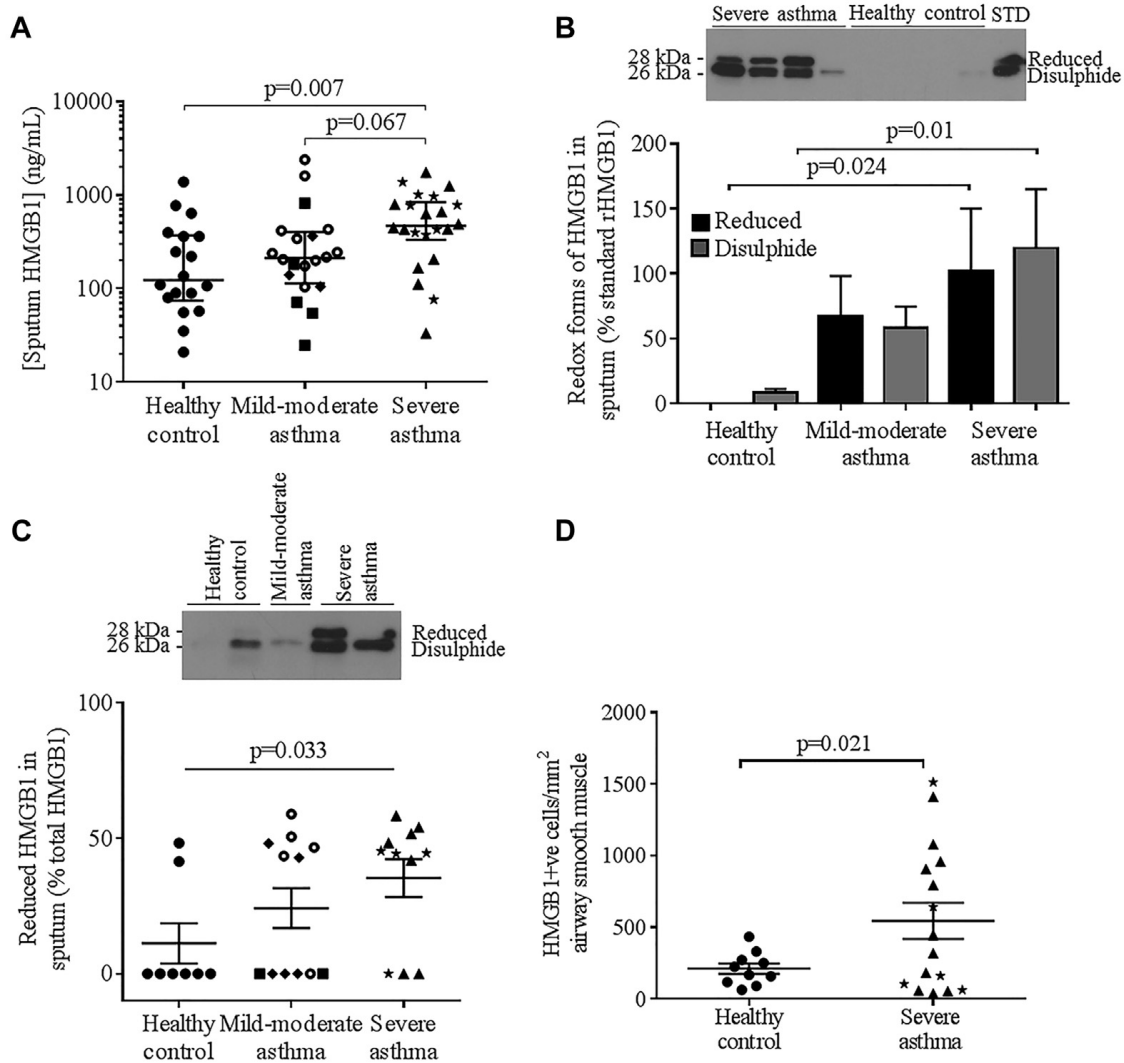
**A,** Sputum HMGB1 concentrations in healthy controls, patients with mild to moderate asthma, and patients with severe asthma (horizontal bar geometric mean and 95% CI). **B,** Disulphide and reduced redox forms of HMGB1 expressed as mean ± SEM % of standard rHMGB1 (STD), sputum from healthy controls (n = 6), patients with mild to moderate asthma (n = 4), and patients with severe asthma (n = 5) and with a representative Western blot above. **C,** Relative expression of reduced versus disulphide HMGB1 in sputum with detectable levels of HMGB1 from healthy controls (n = 8), patients with mild to moderate asthma (n = 12), and patients with severe asthma (n = 11), with a representative Western blot above. **D,** HMGB1^+^ cells/mm^2^ ASM in bronchial biopsies. Symbol key: ● = healthy control; ■ = GINA 1; ◆ = GINA 2;  = GINA 3; ▲ = GINA 4; ★ = GINA 5. *GINA*, Global Initiative for Asthma.

HMGB1 expression was investigated in primary ASMCs. Although there was no differential expression of HMGB1 mRNA (Fig 2, *A*), HMGB1 protein expression was significantly reduced in ASMCs from those with asthma versus controls, assessed by flow cytometry (Fig 2, *B*) and immunofluorescence (see Fig E2, *A* and *B*, in this article's Online Repository at www.jacionline.org). Release of HMGB1 extracellularly by ASMCs from those with asthma and/or the absence in culture of proinflammatory mediators present in asthmatic airways could explain the anomalous *in vitro* and *in vivo* data. Indeed HMGB1 protein expression was significantly upregulated in ASMCs from those with asthma but not controls following stimulation with TNF-α, IL-1β and IFN-γ, or poly(I:C) (Fig 2, *C*); however, HMGB1 expression poststimulation was not different between asthma and health. HMGB1 in ASMC supernatants was below the limit of detection.

**Fig 2:**
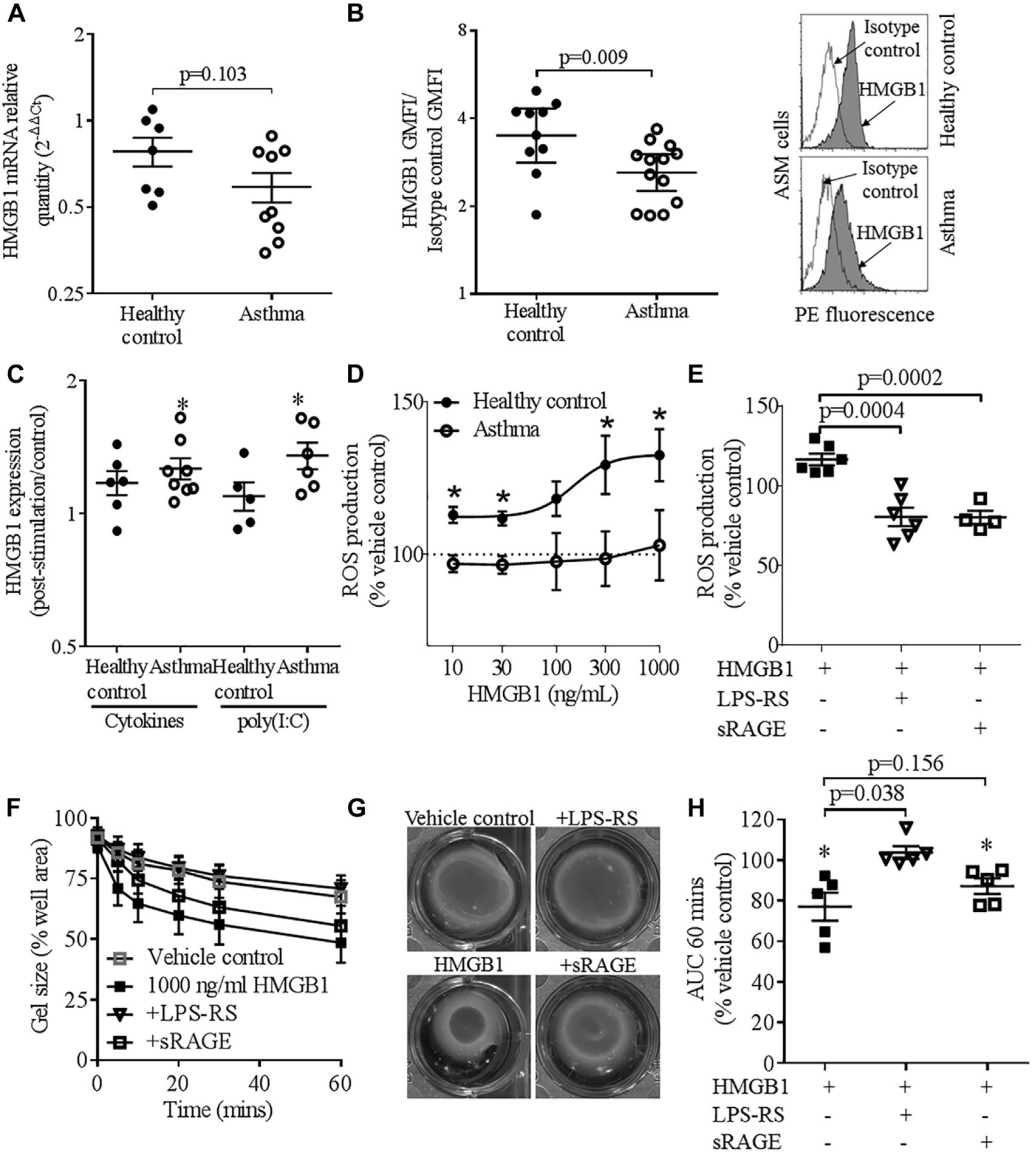
HMGB1 mRNA and protein expression in ASMCs assessed by **(A)** quantitative PCR and flow cytometry **(B)** at baseline with representative histograms to the right and **(C)** following stimulation with proinflammatory cytokines (TNF-α, IFN-γ, and IL-1β, 10 ng/mL) or the dsRNA mimic poly(I:C) (12.5 μg/mL). *P < .05 versus unstimulated ASMCs. ROS production in response to **(D)** HMGB1 (10-1000 ng/mL) in ASMCs, *P ≤ .05 healthy control (●) vs patient with asthma (■), unpaired *t* test, (n = 5-9 ASM donors), and **(E)** HMGB1 (1000 ng/mL) ± LPS-RS or sRAGE (10 μg/mL) in ASMCs from healthy controls. *P* values from unpaired *t* tests. **F-H,** Contraction of collagen gels impregnated with ASMCs from subjects with asthma in the presence of bradykinin following incubation with vehicle control or 1000 ng/mL HMGB1 ± LPS-RS or sRAGE (10 μg/mL) expressed as (Fig 2, *F*) time course, *P < .05 vs vehicle control, (Fig 2, *G*) example collagen gels at 60 minutes, and (Fig 2, *H*) AUC at 60 minutes. *P < .05 vs vehicle control, paired *t* tests. Means ± SEM are shown. *GMFI*, Geometric mean fluorescence intensity; *PE*, phycoerythrin.

Reduced recombinant HMGB1, at concentrations equivalent to those in sputum, caused a concentration-dependent increase in intracellular ROS production in ASMCs from controls, but not in ASMCs from those with asthma (Fig 2, *D*), which was reduced by the RAGE decoy receptor soluble RAGE (sRAGE) and the Toll-like receptor (TLR) 4 antagonist LPS from *Rhodobacter sphaeroides* (LPS-RS) (Fig 2, *E*). This defective response in ASMCs from those with asthma was not due to impaired ROS generation capacity; ROS production was similar at baseline, and was increased in response to hydrogen peroxide stimulation of ASMCs from those with asthma versus controls.^5^ In addition, ASMC RAGE and TLR4 expression was not different between those with asthma and controls (Fig E2, *C-E*). HMGB1 activity and receptor binding is dependent on its oxidation state, and is regulated by several binding partners.^4^ The complex interplay between these factors affects the cellular response to HMGB1 and might affect ROS production in ASM from those with asthma.

In the absence of bradykinin, contraction of collagen gels impregnated with ASMCs from those with asthma was not significantly different from controls (see Fig E3, *A*, in this article's Online Repository at www.jacionline.org), nor was it affected by HMGB1 (100, 300 [data not shown], and 1000 ng/mL [Fig E3, *B* and *C*]). However bradykinin-mediated contraction of collagen gels impregnated with ASMCs was potentiated by 1000 ng/mL HMGB1, resulting in a decrease in the area under the curve at 60 minutes (Fig E3, *D* and *E*), but not 100 to 300 ng/mL HMGB1 (data not shown). In the presence of HMGB1, bradykinin-mediated contraction of collagen gels impregnated with ASMCs from those with asthma was potentiated to a greater extent compared with controls (Fig E3, *F*) and was significantly inhibited by LPS-RS, but not by sRAGE (Fig 2, *F-H*). sRAGE and LPS-RS had no significant effect in the absence of HMGB1.

HMGB1 and/or sRAGE or LPS-RS (10 μg/mL) had no effect on ASMC migration (wound healing assay: HMGB1 [100-1000 ng/mL] ± CXCL12 [10-100 ng/mL], data not shown, ORIS assay: 3-1000 ng/mL HMGB1, Fig E3, *G*), proliferation in the presence/absence of serum (Fig E3, *H*), and apoptosis or necrosis (Fig E3, *I* and *J*).

Our data support and extend previous studies suggesting an imbalance between HMGB1 and endogenous secretory RAGE in the asthmatic airways might have implications for HMGB1 in asthma pathophysiology.^6^ Because the increased HMGB1 we see in the sputum in asthma correlates with sputum total cell and nonviable cell counts, we propose that HMGB1 can be upregulated in the airways in asthma because of inflammatory and stress stimuli that can result in HMGB1 secretion actively by activated immune cells and passively by necrotic cells.^7^, ^8^

We propose that the ROS produced in response to HMGB1 in ASMCs from controls, in a RAGE/TLR4-dependent manner, terminally oxidize HMGB1, rendering it inactive^4^ or alter Ca^2+^ homeostasis, leading to reduced contractility via a TLR4/ROS-dependent mechanism as in murine cardiomyocytes,^9^ thus limiting the potentiation of contraction of collagen gels impregnated with ASMCs from controls. Because of the ROS-generating capacity of ASMCs in response to HMGB1 being defective in asthma, these ROS-mediated responses would be reduced. Therefore, HMGB1 can potentiate contraction of collagen gels impregnated with ASMCs from those with asthma, in a TLR4-dependent manner, to a greater extent than those impregnated with ASMCs from controls. Thus, HMGB1 could contribute to ASM dysfunction and airway hyperresponsiveness in asthma, as supported by animal models,^3^, ^10^ possibly representing a potential therapeutic target.

## Figures and Tables

**Fig E1:**
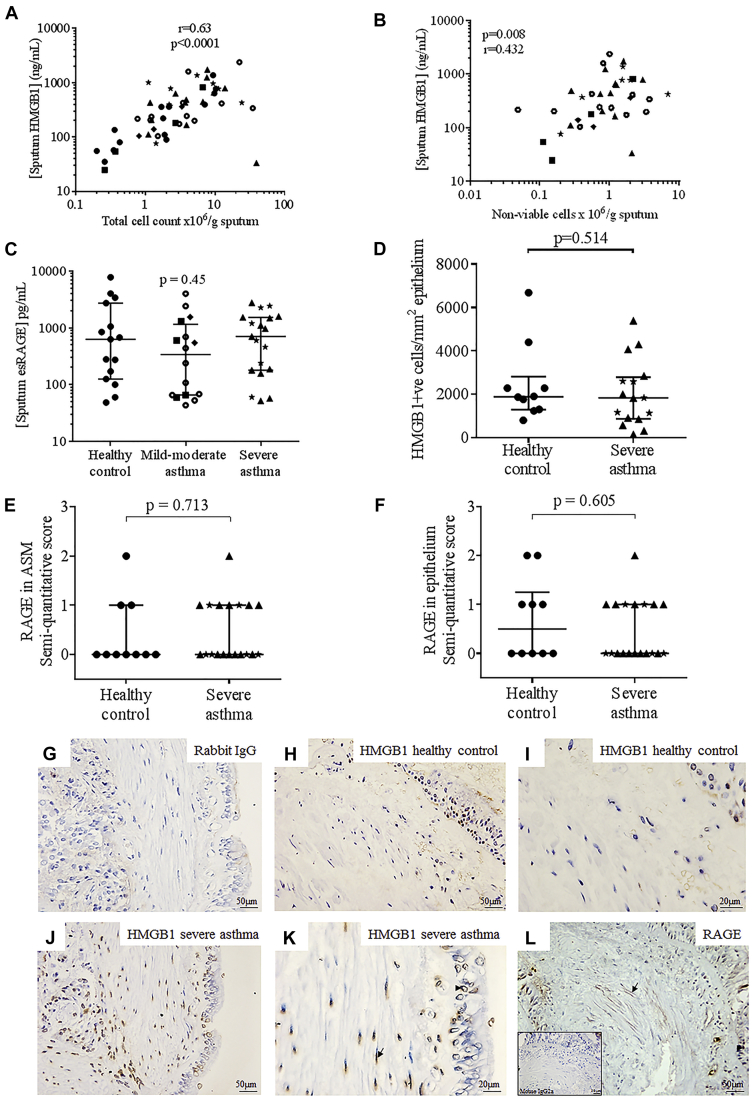
Sputum HMGB1 correlated with sputum total cell count (**A**) and sputum nonviable cell count (**B**) in patients with asthma. **C,** Sputum esRAGE concentration in subjects with asthma and healthy controls. Horizontal bar represents geometric mean (95% CI). *P* value from Kruskall-Wallis test. Subject characteristics are presented in Table E2. **D,** The number of HMGB1^+^ cells/mm^2^ epithelium in bronchial biopsies. **E** and **F,** RAGE expression was assessed using a semi-quantitative scoring system: 0 = no positive staining; 1 = little positive staining; 2 = moderate positive staining; 3 = marked positive staining by a blinded observer in (Fig E1, *E*) ASM and (Fig E1, *F*) epithelium in bronchial biopsies. **G-L,** HMGB1/RAGE staining in bronchial tissue: *G*, Isotype control (rabbit immunoglobulin fraction, ×200 magnification), HMGB1 staining (brown) in a healthy control (*H*, ×200 magnification; *I*, ×400 magnification) and in a subject with severe asthma (*J*, ×200 magnification; *K*, ×400 magnification); positive staining was observed in the smooth muscle *(arrow)* and the epithelium (arrow head). **L,** RAGE staining in a tissue section from a patient with severe asthma showing RAGE expression in smooth muscle *(arrow)* and epithelium *(arrowhead)* at ×200 magnification. Inset: mouse IgG_2a_ isotype control (×200 magnification). Subject characteristics are presented in Table E3. Symbol key: ● = healthy control; ■ = GINA 1; ◆ = GINA 2;  = GINA 3; ▲ = GINA 4; ★ = GINA 5. *esRAGE*, Endogenous secretory RAGE.

**Fig E2:**
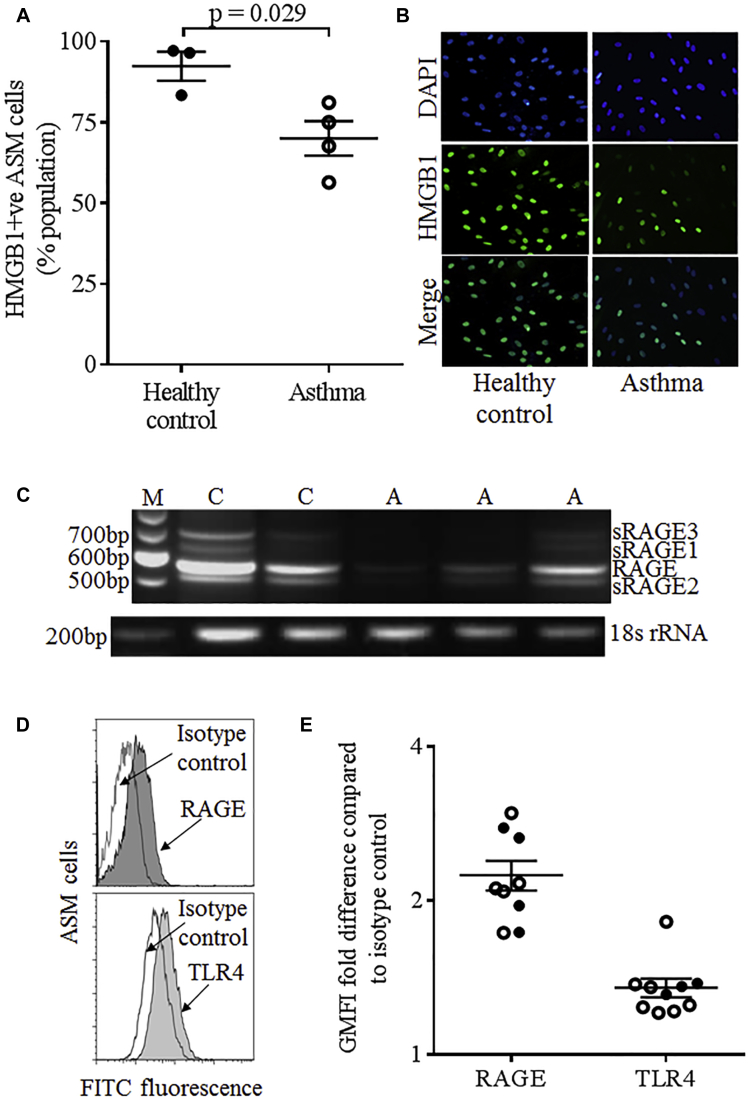
**A,** HMGB1 protein expression in ASM cells was assessed by immunofluorescence. **B,** Representative immunofluorescent staining of cell nuclei (DAPI, *upper panel*), HMGB1 *(middle panel)*, and merged DAPI and HMGB1 *(lower panel)*. **C,** ASM from subjects with asthma and control subjects was shown to express different isoforms of RAGE by PCR. PCR products were isolated by gel purification, sequenced, and identified as full-length membrane-bound RAGE (RAGE), or soluble isoforms of RAGE (sRAGE 1-3). *M* = molecular weight marker, *C* = healthy control, *A* = asthma. **D,** Representative flow cytometry histograms showing cell-surface RAGE ( FITC fluorescence, *upper panel*) and TLR4 (Alexa Fluor 488 fluorescence, *lower panel*) expression in ASM cells. *Open histograms* represent isotype controls; *dark gray* and *light gray shaded histograms* are RAGE or TLR4-stained cells, respectively. **E,** ASM cell-surface receptor expression was expressed as gMFI (fold change antibody/isotype control) for healthy control subjects (●) and subjects with asthma () for RAGE or TLR4 (*P* = .84 and *P* = .99, healthy control vs patient with asthma, respectively, unpaired *t* test). *DAPI*, 4′,6′-Diamidino-2 phenylindole; *FITC*, fluorescein isothiocyanate.

**Fig E3:**
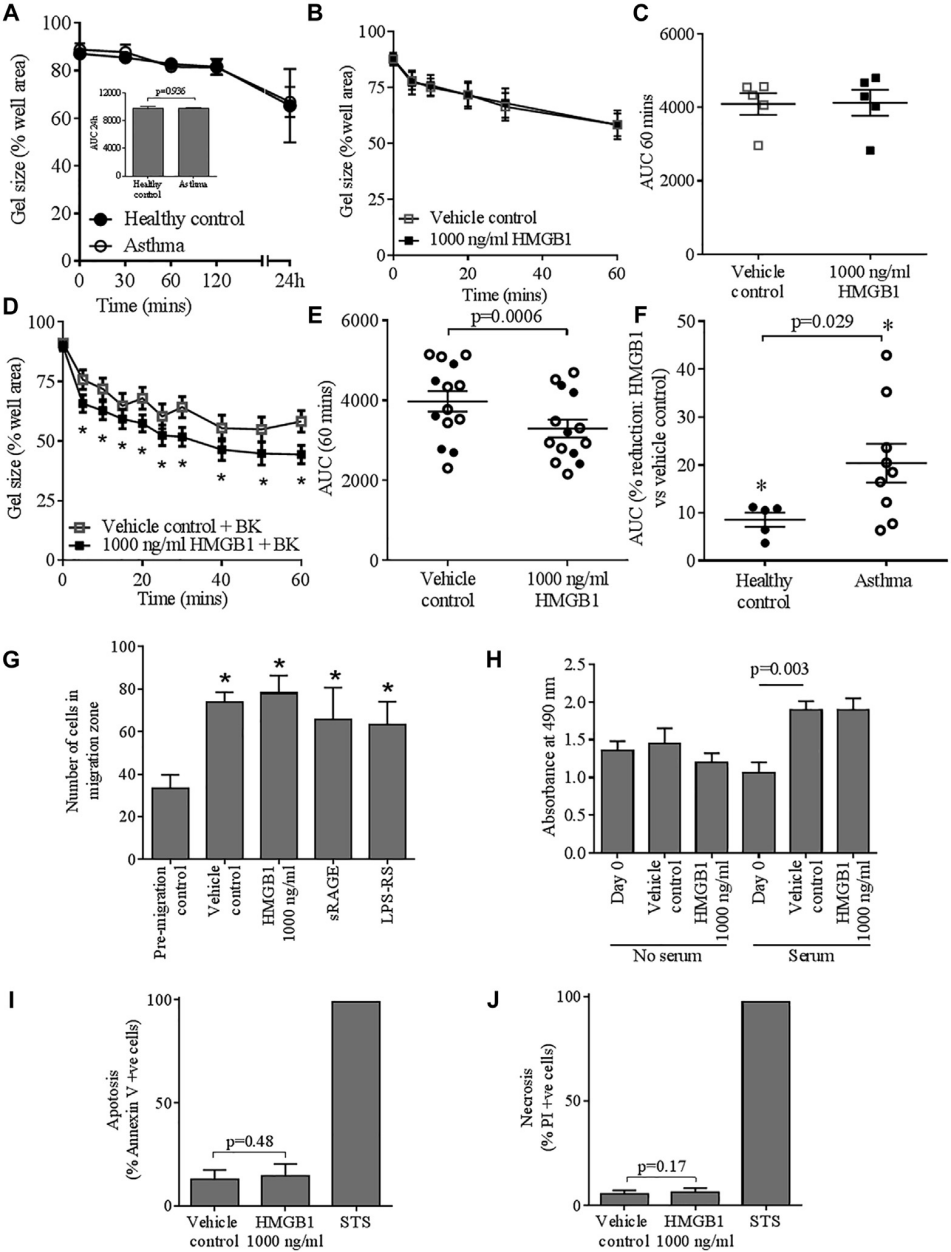
**A,** Time course of contraction of collagen gels impregnated with ASM cells from healthy controls and subjects with asthma in the absence of bradykinin, with the area under the curve (AUC) over a 24-hour period as an inset. Time course (**B**) and AUC over a 60-minute period (**C**) of contraction of collagen gels impregnated with ASM from subjects with asthma in the absence of bradykinin, following incubation with HMGB1 (1000 ng/mL, n = 5). **D,** Time course of contraction of collagen gels impregnated with ASM cells in the presence of bradykinin following incubation with HMGB1 (1000 ng/mL), n = 12 ASM donors, *P < .05 HMGB1 versus vehicle control, paired *t* test at each time point. **E,** AUC over a 60-minute period of contraction of collagen gels impregnated with ASM cells in the presence of bradykinin following incubation with HMGB1 (1000 ng/mL), healthy control (●), asthma (), *P* < .05, paired *t* test. **F,** Reduction in AUC at 60 minutes (% vehicle control) in gels impregnated with ASM derived from both healthy controls and subjects with asthma, *P < .05 versus vehicle control. **G,** Compared with the premigration control significant ASM cell migration, measured by counting Hoechst-positive cell nuclei in the migration zone, was seen (*P < .05 vs premigration control) but was not significantly different following treatment for 24 hours with exogenous 1000 ng/mL HMGB1, or by blocking the activity of endogenously released HMGB1 with 10 μg/mL sRAGE or 10 μg/mL LPS-RS. **H,** Cell proliferation as measured by the CellTiter AQueous One Solution Cell Proliferation Assay was unaffected following treatment with 1000 ng/mL HMGB1 for 72 hours in the presence or absence of serum. The percentage of cells undergoing apoptosis as detected by Annexin V staining **(I)** and necrosis as detected by propidium iodide **(J)** was not significantly different following treatment with 1000 ng/mL HMGB1. Staurosporine (STS) was used as a positive control.

**Table E1:** Subjects' characteristics for the HMGB1 ELISA

Characteristic	Healthy control (n = 18)	Asthma mild-moderate (n = 20)	Asthma severe (n = 22)	ANOVA/χ^2^ test∗
GINA classification, n		GINA 1, n = 5 GINA 2, n = 3 GINA 3, n = 12	GINA 4, n = 13 GINA 5, n = 9	
Age (y)†	53.0 ± 3.2	57.6 ± 3.2	57.3 ± 2.6	*P* = .51
Sex: male, n (%)	7 (39)	12 (60)	11 (50)	*P* = .43∗
Atopy, n (%)	4 (22.2)	12 (60)	17 (77)	*P* = .002∗
Smoking status, current smoker, n (%)	0 (0)	1 (5)	3 (14)	*P* = .21∗
ICS (μg/d BDP equivalent)‡	0 (0)	200 (400)§	1600 (400)§||	*P* < .001
Pre-BD FEV_1_ % predicted‡	91.5 (35.6)	87.0 (25.0)	72.5 (41.5)§	*P* = .03
Pre-BD FEV_1_/FVC‡	74.5 (6.0)	73.0 (10.5)	72.0 (15.0)	*P* = .21
% Sputum neutrophils†	59.8 ± 5.3	58.1 ± 6.6	62.7 ± 4.5	*P* = .88
% Sputum eosinophils‡	0.75 (1.4)	1.0 (3.3)	2.8 (13.9)§||	*P* = .002
% Sputum epithelial cells‡	1.60 (2.3)	2.25 (4.8)	1.4 (1.7)	*P* = .735

*BDP*, Beclometasone dipropionate; *FVC*, forced vital capacity; *ICS*, inhaled corticosteroid; *pre-BD*, prebronchodilator.

∗ χ^2^ test.

† Mean ± SEM.

‡ Median (interquartile range).

§ *P* < .05 vs healthy control.

|| *P* < .05 vs mild asthma.

**Table E2:** Subjects' characteristics for esRAGE ELISA

Characteristic	Healthy control (n = 15)	Asthma mild-moderate (n = 16)	Asthma severe (n = 19)	ANOVA/χ^2^ test∗
GINA classification, n		GINA 1, n = 4 GINA 2, n = 2 GINA 3, n = 10	GINA 4, n = 12 GINA 5, n = 7	
Age (y)†	53.1 ± 3.9	58.2 ± 3.7	59.6 ± 2.5	*P* = .37
Sex: male, n (%)	7 (46.7)	8 (50)	10 (52.6)	*P* = .94∗
Atopy, n (%)	4 (26.7)	9 (56.25)	15 (78.9)	*P* = .01∗
Smoking status, current smoker, n (%)	0 (0)	1 (6.25)	3 (15.8)	*P* = .23∗
ICS (μg/d BDP equivalent)‡	0 (0)	200 (400)	2000 (400)§||	*P* < .001
Pre-BD FEV_1_ % predicted‡	94.0 (50.5)	86.5 (32.3)	75.0 (16.0)§	*P* = .030
Pre-BD FEV_1_/FVC‡	75.0 (6.0)	73.5 (13.8)	72.0 (14.0)	*P* = .09
% Sputum neutrophils†	62.3 ± 5.8	59.5 ± 7.0	59.4 ± 4.6	*P* = .93
% Sputum eosinophils‡	0.75 (1.96)	0.75 (1.75)	4.25 (13.3)§||	*P* = .001
% Sputum epithelial cells‡	1.2 (2.1)	1.5 (4.8)	1.5 (3.0)	*P* = .90

Samples used are subsets of those used for the HMGB1 ELISA.

*BDP*, Beclometasone dipropionate; *esRAGE*, endogenous secretory RAGE; *ICS*, inhaled corticosteroid; *FVC*, forced vital capacity; *pre-BD*, prebronchodilator.

∗ χ^2^ test.

† Mean ± SEM.

‡ Median (interquartile range).

§ *P* < .05 vs healthy control.

|| *P* < .05 vs mild asthma.

**Table E3:** Subjects' characteristics for immunohistochemistry

Characteristic	Healthy control (n = 10)	Asthma severe (n = 19)	*t* test/χ^2^ test∗
GINA classification, n		GINA 4, n = 11 GINA 5, n = 5	
Age (y)†	46.7 ± 6.2	55 ± 2.8	*P* = .17
Sex: male, n (%)	5 (50)	8 (42.1)	*P* = .71∗
Smoking status, current smoker, n (%)	1 (10)	5 (26.3)	*P* = .63∗
ICS (μg/d BDP equivalent)‡	0 (0)	1600 (125)§	*P* < .001
Pre-BD FEV_1_ % predicted‡	93.5 (28.6)	76.0 (54.7)	*P* = .065
Pre-BD FEV_1_/FVC‡	90.5 (13.3)	71.1 (24.9)§	*P* = .0009
% Sputum neutrophils†	50.5 ± 18.7	52.6 ± 6.1	*P* = .9
% Sputum eosinophils‡	0.4 (0.8)	5.3 (16.8)§	*P* = .002

*BDP*, Beclometasone dipropionate; *ICS*, inhaled corticosteroids; *FVC*, forced vital capacity; *pre-BD*, pre-bronchodilator.

∗ χ^2^ test.

† Mean ± SEM.

‡ Median (interquartile range).

§ *P* < .05 vs healthy control.

## Methods

### Subjects

Subjects were recruited from Glenfield Hospital, Leicester, United Kingdom. Asthma severity was defined according to the Global Initiative for Asthma (GINA) treatment steps^E1^: mild to moderate asthma, GINA 1 to 3; severe asthma, GINA 4 to 5. Healthy subjects had no history of respiratory disease and normal spirometry. The study was approved by the Leicestershire Research Ethics Committee. Informed consent was obtained from all subjects (UHL 10613).

### Sputum induction

Sputum was induced using inhaled incremental concentrations of nebulized hypertonic saline at 3%, 4%, and 5%, each administered for 5 minutes. Sputum plugs were selected and diluted in 8 volumes (weight/volume) of PBS. Samples were rocked on ice for 15 minutes and then spun at 790*g* for 10 minutes at 4°C. Four volumes of the PBS supernatants were collected and kept at −80°C until further use (eg, HMGB1 ELISA and Western blotting). The remaining cell and mucus pellets were resuspended in 4 volumes of 0.2% dithiothreitol (DTT) and rocked on ice for 15 minutes to disperse the cells. The cell suspension was then filtered through a 48-μm nylon gauze and the total cell number was determined using a hemocytometer. The DTT supernatant was obtained by centrifugation as before, removed, and stored at –80°C. Cells were resuspended in PBS at a density of 0.5 to 0.75 × 10^6^ per mL and 75 μL of the cell suspension was adhered onto glass slides using cytospins (450 rpm for 6 minutes). Slides were air-dried for at least 15 minutes, fixed with 90% methanol for 10 minutes, and then stained with the Romanowsky method for differential cell counts to measure the percentage of sputum neutrophils, eosinophils, macrophages, epithelial cells, and lymphocytes.^E2^

### ELISA

HMGB1 was detected using a kit from Oxford Biosystems (Milton Park, Oxfordshire, United Kingdom) according to manufacturer's instructions. All redox forms of HMGB1 are detected. Endogenous secretory RAGE was detected using a kit from B-Bridge International (distributed by Metachem Diagnostics Ltd, Northampton, United Kingdom) according to manufacturer's instructions.

### Western blotting

An equivalent amount of each PBS-diluted sputum sample was loaded on gels. SDS-PAGE was performed on a 12% gel under nonreducing conditions to preserve the redox status of HMGB1 and proteins transferred to nitrocellulose membranes, which were blocked with 5% skimmed milk in Tris-buffered saline, pH 7.0, containing 0.1% Tween 20 (TBS-T). Blocked membranes were probed with rabbit anti-HMGB1 (1:1000) in TBS-T plus 5% milk overnight at 4°C, washed several times with TBS-T, and incubated for 1 hour with antirabbit peroxidase-conjugated antibody (1:10,000). Western blots were visualized using an enhanced chemiluminescence kit according to the manufacturer's instructions (GE Healthcare, Little Chalfont, Buckinghamshire, United Kingdom). Recombinant HMGB1 was run as a standard in a subset of samples to assess the amount of HMGB1 in healthy controls versus samples with asthma. Data were analyzed by densitometry and normalized to a recombinant HMGB1 standard for differences between sputa from healthy controls and patients with asthma, or in samples with detectable HMGB1 the amount of reduced HMGB1 was calculated as a percentage of total HMGB1 in the sample.

### Immunohistochemistry

Human bronchial biopsies were embedded into glycol methacrylate (Polysciences, Northampton, United Kingdom). Two-micrometer sections were probed with a mouse monoclonal alpha-smooth muscle actin antibody (clone 1A4, Dako, Cambridge, United Kingdom) or appropriate isotype control, to identify ASM. Sequential sections were stained with a rabbit monoclonal anti-HMGB1 antibody (20 μg/mL, clone EPR3507, Abcam, Cambridge, United Kingdom), a mouse monoclonal anti-RAGE antibody (Millipore, Watford, United Kingdom, clone DD/A11, 10 μg/mL), or appropriate isotype controls (Dako). For RAGE staining, an amplification step was performed with a mouse LINKER for 15 minutes at room temperature. The Envision FLEX kit (Dako) was used. Nuclei were identified with Mayer's hematoxylin (blue staining). Positive staining was quantified per mm^2^ smooth muscle or epithelial area (HMGB1) or by semi-quantitative scoring for RAGE (0 = no positive staining, 1 = little positive staining, 2 = moderate positive staining, 3 = marked positive staining) by a blinded observer.

### Cell culture

ASM bundles were isolated from bronchial biopsies. Primary ASM cells were cultured in Dulbecco modified Eagle medium (DMEM) with Glutamax-1 supplemented with 10% FBS, 100 U/mL penicillin, 100 μg/mL streptomycin, 0.25 μg/mL amphotericin, 100 μM nonessential amino acids, and 1 mM sodium pyruvate (Gibco, Paisley, United Kingdom). Cells were characterized for αSMA expression by flow cytometry. ASM cells from patients with asthma from a range of GINA treatment steps were used with no noticeable differences in response to HMGB1 stimulation (*P* < .05 where n numbers are sufficient for statistical analysis).

### RT-PCR

RT-PCR was performed to investigate the splice variants of RAGE (accession no. M91211, Genbank) expressed by ASM cells, using a primer pair previously used^E3^ to simultaneously detect soluble and transmembrane RAGE forms: RAGE F: GATCCCCGTCCCACCTTCTCCTGTAGC and RAGE R: CACGCTCCTCCTCTTCCTCCTGGTTTTCTG from Invitrogen, Paisley, United Kingdom. 18S rRNA (accession no. NR_003286, Genbank) was also amplified as a loading control. 18sRNA primers were from Invitrogen, h18SRNA.891F: GTTGGTTTTCGGAACTGAGG (forward) and h18SRNA.1090R: GCATCGTTTATGGTCGGAAC (reverse). PCR products were run on an agarose gel, gel purified, and sequenced (Protein/Nucleic Acid Chemistry Laboratory, University of Leicester, Leicester, United Kingdom).

### Quantitative PCR

Reverse transcription real-time PCR was used for relative quantification of mRNA expression of HMGB1 in ASM cell culture derived from normal and asthma donors. Briefly, total cellular RNA was isolated from cultured ASM cells using Peq Gold total RNA kit (Peq Lab) and on membrane DNaseI treatment according to manufacturer's instructions. RNA quality and quantity were assessed using a TECAN infinite NANO-QUANT plate reader (Tecan, Reading, United Kingdom) and 1 μg of total RNA from each ASM cell culture was reverse transcribed using SuperScript Vilo cDNA synthesis kit (Invitrogen). Amplification of 10 ng of cDNA per reaction in a final volume of 20 μL was performed using the Express SYBR GreenER qPCR SuperMix Universal (Invitrogen) in a Chromo4 Real-Time Detector (Bio-Rad, Watford, United Kingdom). All samples were tested in triplicate and 18s RNA was used for normalization. HMGB1 primers were from Primerdesign Ltd (Southampton, United Kingdom), HMGB1 F: GTGCAAAGGTTGAGAGCTATTG and HMGB1 R: AATAAATACAGCAAACATTAACAACAC. 18sRNA primers were used as for RT-PCR. The relative quantification was done using the comparative 2^−ΔΔCt^ method and expressed as arbitrary units. For each individual donor, the ΔCt was calculated from the Ct HMGB1 individual donor − Ct 18sRNA individual donor. The calibrator ΔCt is the average ΔCt of the healthy controls. Thus, the ΔΔCt of each individual donor = ΔCt of each individual donor − calibrator ΔCt. PCR products were run on an agarose gel, gel purified, and sequenced (Protein/Nucleic Acid Chemistry Laboratory, University of Leicester).

### Immunofluorescence

ASM cells were fixed and stained with a mouse monoclonal anti-HMGB1 antibody (10 μg/mL, clone 2F6, Abnova, Lutterworth, United Kingdom). Secondary antibody was rabbit antimouse Alexa Fluor 488 (Invitrogen). Cells were counterstained with 4′,6′-diamidino-2 phenylindole (1 μg/mL; Sigma, Gillingham, Dorset, United Kingdom) to identify nuclei. An Olympus BX50 fluorescent microscope mounted with an Olympus DP72 camera was used to visualize staining. At least 3 fields of view with at least 14 cells per field of view were assessed per donor. HMGB1 staining in ASM cells was predominantly nuclear; thus, the number of HMGB1 +ve cells was expressed by calculating the number of HMGB1-positive nuclei as a percentage of ASM nuclei identified by 4′,6′-diamidino-2 phenylindole staining.

### Flow cytometry

Cell-surface protein expression was measured in nonpermeabilized ASM cells using mouse mAbs: anti-RAGE (12 μg/mL, clone A11, Santa Cruz Biotechnology, Heidelberg, Germany) and anti-TLR4 Alexa Fluor 488 (15 μg/mL, clone HTA125, eBioscience, Hatfield, United Kingdom). Secondary antibody for RAGE was rabbit antimouse fluorescein isothiocyanate (Dako). HMGB1 was measured in ASM cells permeablized with PBS containing 0.5% BSA and 0.1% saponin, using the anti-HMGB1 antibody (200 μg/mL, clone EPR3507, Abcam) and a sheep antirabbit R-phycoerythrin antibody (AbD Serotec, Oxford, United Kingdom). Isotype controls were rabbit immunoglobulin fraction (Dako), mouse IgG_2a_ (Dako), and mouse IgG_2a_ κ Alexa Fluor 488 (eBioscience). For some experiments, ASM cells were incubated in serum-free DMEM plus or minus proinflammatory cytokines (recombinant human TNF-α, IFN-γ, IL-1β, 10 ng/mL, R&D Systems, Oxford, United Kingdom) or poly(I:C) (12.5 μg/mL, Sigma-Aldrich, Gillingham, United Kingdom) for 16 hours. One-color flow cytometry was performed with a FACSCanto (BD Biosciences, Oxford, United Kingdom) and analysis performed with FlowJo software.

### Intracellular ROS production

Intracellular ROS production was measured using 2′,7′-dichlorodihydrofluorescein diacetate (H_2_DCFDA, Invitrogen). Quadruplicate repeats for vehicle control or reduced recombinant HMGB1 (provided in PBS, 1 mM EDTA, 1 mM DTT, pH 7.2 solution, R&D Systems, Oxford, United Kingdom, endotoxin level: <0.10 EU per 1 μg of protein [LAL method]) were added in phenol red-free DMEM. Fluorescence (excitation 493 nm, emission 523 nm) was measured with an EnSpire multimode plate reader (Perkin Elmer, Cambridge, United Kingdom). For some experiments, ASM cells were preincubated with sRAGE (10 μg/mL, Aviscera Bioscience, Cambridge, United Kingdom) or ultrapure LPS from *Rhodobacter sphaeroides* (LPS-RS Ultrapure, 10 μg/mL, InvivoGen, Toulouse, France) for 1 hour. HMGB1 binding to 10 μg/mL sRAGE does not reach saturation at 1000 ng/mL HMGB1^E4^ and an HMGB1:RAGE ratio of approximately 1:1 has been shown to inhibit HMGB1 activity by 75%.^E5^ LPS-RS was used at a ratio of 10:1 exceeding that previously shown to antagonize HMGB1 activation of TLR4.E6, E7

### Collagen gel contraction assay

Collagen gel contraction was assessed as described previously.^E8^ The collagen gel mixture (299 μL PureCol, 3 mg/mL, 37 μL 10 DMEM, 144 μL ASM cell suspension in serum-free DMEM, 20 μL sodium bicarbonate, 7.5%) was impregnated with ASM cells in the presence of vehicle control or reduced recombinant HMGB1 (300-1000 ng/mL) in the presence or absence of sRAGE or LPS-RS (both at 10 μg/mL) and allowed to set for 90 minutes. Gels were detached from the well using a spatula and gel contraction measured in the absence or presence of the contractile stimulus bradykinin (1 ng/mL, Sigma-Aldrich, Gillingham, United Kingdom). Images were captured over a 1-hour period and gel area was measured as a percentage of well area using ImageJ software.

### Cell migration

#### Oris cell migration assay

ASM migration was assessed using the Oris cell migration kit (Tebu-bio, Peterborough, United Kingdom). Quadruplicate repeats were performed for premigration control, vehicle control, reduced recombinant HMGB1 (1000 ng/mL, R&D Systems), sRAGE (10 μg/mL), or LPS-RS (10 μg/mL), which were added for 24 hours in serum-free DMEM. Cells were fixed and stained with Hoechst nuclear dye (Invitrogen). Images were captured with an EVOS fluorescence microscope (Invitrogen Life Technologies, Paisley, United Kingdom) and the number of cells in the migration zone compared with the premigration control was counted.

#### Wound healing assay

ASM cells were grown to confluence in 6-well plates coated with 10 μg/mL fibronectin to ensure cell adherence during wounding, serum deprived for 24 hours, and then wounded using a sterile 200 μL pipette tip in a predetermined grid pattern. Following wounding, ASM cells were washed 4× with serum-free media before the addition of serum-free media HMGB1 (100-1000 ng/mL) ± CXCL12 (10-100 ng/mL). Wounds were then photographed at baseline and after 24 hours. The number of cells that had moved into the wound after 24 hours was counted by a blind observer.

#### CellTiter 96 AQueous One Solution cell proliferation assay

Serum-free medium ± 1000 ng/mL reduced recombinant HMGB1 was added to confluent ASM cells for 24 hours. The CellTiter 96 AQueous One solution with the tetrazolium compound (3-(4,5-dimethylthiazol-2-yl)-5-(3-carboxymethoxyphenyl)-2-(4-sulfophenyl)-2H tetrazolium inner salt [Promega, Southampton, United Kingdom]) was then added to each well according to manufacturer's instructions and the absorbance at 490 nm read on an EnSpire plate reader (Perkin Elmer).

### Apoptosis and necrosis

The percentage of apoptotic and necrotic ASM cells exposed to serum-free medium ± 1000 ng/mL reduced recombinant HMGB1 for 24 hours was identified by staining with fluorescein isothiocyanate–conjugated Annexin V (1 μL/200 μL binding buffer, Bioscience) ± propidium iodide (0.5 μg/mL, Bioscience), before acquisition on a flow cytometer and analysis using FlowJo software.

### Data analysis

Statistical analysis was performed using GraphPad Prism 6.0 (San Diego, Calif). Parametric data were analyzed with paired or unpaired *t* tests or ANOVA followed by appropriate *post hoc* multiple comparison tests. Nonparametric data were analyzed using Kruskal-Wallis test followed by Dunn's post hoc test. Spearman correlations were used. Categorical data were analyzed with χ^2^ test. *P* ≤ .05 was considered significant.
